# Test, test, test – a complication of testing for coronavirus disease 2019 with nasal swabs

**DOI:** 10.1017/S0022215120001425

**Published:** 2020-07-09

**Authors:** Z Mughal, E Luff, O Okonkwo, C E J Hall

**Affiliations:** Department of ENT and Head and Neck Surgery, Gloucestershire Hospitals NHS Foundation Trust, Gloucestershire Royal Hospital, Gloucester, UK

**Keywords:** Nose, Foreign Bodies, Coronavirus

## Abstract

**Background:**

Coronavirus disease 2019, a highly transmissible respiratory infection, has created a public health crisis of global magnitude. The mainstay of diagnostic testing for coronavirus disease 2019 is molecular polymerase chain reaction testing of a respiratory specimen, obtained with a viral swab. As the incidence of new cases of coronavirus disease 2019 increases exponentially, the use of viral swabs to collect nasopharyngeal specimens is anticipated to increase drastically.

**Case report:**

This paper draws attention to a complication of viral swab testing in the nasopharynx and describes the premature engagement of a viral swab breakpoint, resulting in impaction in the nasal cavity.

**Conclusion:**

This case highlights a possible design flaw of the viral swab when used to collect nasopharyngeal specimens, which then requires an aerosol-generating procedure in a high-risk patient to be performed. The paper outlines a safe technique of nasal foreign body removal in a suspected coronavirus disease 2019 patient and suggests alternative testing materials.

## Introduction

Coronavirus disease 2019 (Covid-19) is a respiratory infection in humans that was first identified as an outbreak of pneumonia of unknown cause in Wuhan city, China, in December 2019.^1,2^ The offending pathogen is a novel coronavirus, isolated on 7th January 2020, known as severe acute respiratory syndrome coronavirus 2 (SARS-CoV-2).^2^ The clinical presentation varies from asymptomatic, to a mild upper respiratory infection, to severe viral pneumonia, respiratory failure and death.^2^ The World Health Organization (WHO) declared a Covid-19 pandemic on 11th March 2020.^3^

Diagnostic testing plays a crucial role in outbreak initial detection, containment, effective isolation and eventual resolution.^4^

## Case report

A male nursing home resident in his late seventies presented to the emergency department after an unwitnessed fall, unable to mobilise. He had a background of severe dementia and chronic kidney disease. He was diagnosed with a right-sided neck of femur fracture. He was admitted and underwent an uncomplicated right-sided hemi-arthroplasty.

Three days post-operatively, he developed a low grade fever. He was suspected of having Covid-19 as he had been in contact with other patients on the ward who had subsequently tested positive for the virus.

The patient lacked capacity and was uncooperative whilst undergoing a nasopharyngeal swab test for Covid-19. Despite gentle restraint from a trained healthcare assistant, rapid head movements resulted in the engagement of the swab breakpoint mechanism, whilst the swab was still in the nasal cavity. The swab was retained in the nose out of direct vision.

**Fig. 1.**
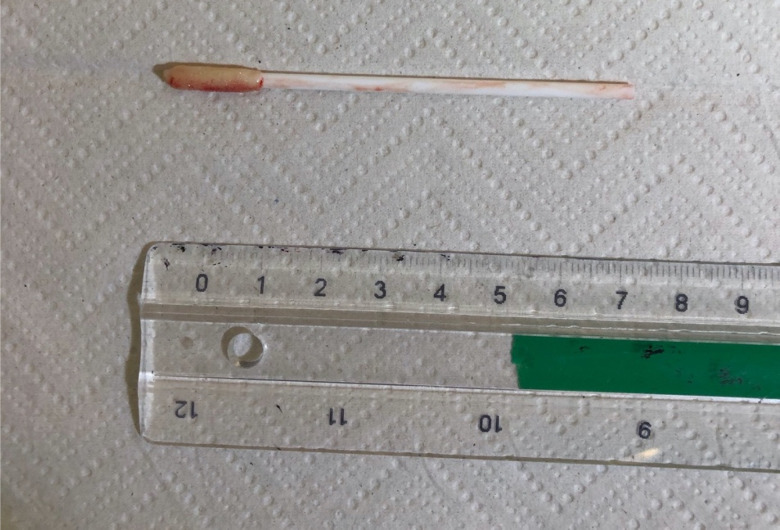
Retained viral swab retrieved from the patient's right nasal cavity.

The ENT registrar was called to retrieve the swab. The patient underwent a bedside examination with a flexible nasendoscope. A nasal swab stick was found in the right nasal cavity, under the inferior turbinate on the floor, midway in the nasal cavity (Figure 1). Retrieval of an 8 cm swab was performed with minimal trauma under direct vision using Tilley's dressing forceps.

The swab tested positive for Covid-19 and the patient was transferred to a ring-fenced Covid-19 ward.

## Discussion

### Testing

The SARS-CoV-2 virus has an affinity for respiratory epithelium. Zou *et al*. compared throat and nasal swabs, and found higher viral loads in the nose compared to the throat.^5^ Liu *et al*. found that patients with severe Covid-19 harboured a greater viral load and a longer virus-shedding period in the nasopharynx compared to mild cases.^6^

The primary means of diagnosing Covid-19 at present is via molecular testing to detect viral RNA using real-time reverse transcriptase polymerase chain reaction.^7^ Clinical suspicion and several laboratory findings may raise the probability of Covid-19, including decreased albumin, raised C-reactive protein, high lactate dehydrogenase, lymphopenia, high aspartate transaminase and high erythrocyte sedimentation rate.^8^ Chest radiographs show bilateral changes, often ground-glass opacification.^8^ Computed tomography changes include multifocal bilateral or isolated round ground-glass opacity with or without patchy consolidations, prominent peripherally and in the posterior or lower lobe.^9^

### Nasal swabs

**Fig. 2.**
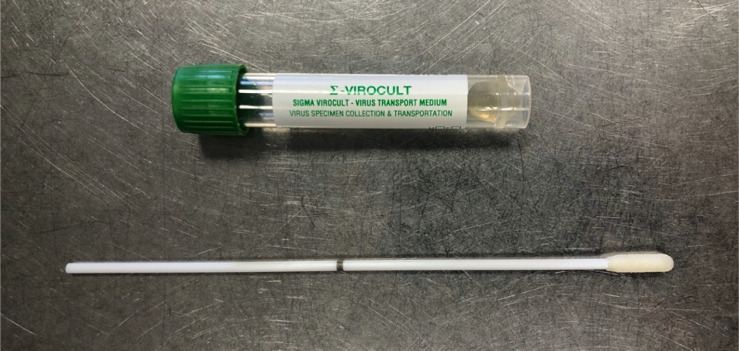
Viral swab with breakpoint mechanism (black line on the swab shaft) and accompanying bottle containing virus transport medium.

The viral specimen swabs used in our unit are Sigma Virocult®.^10^ They consist of two components, a plastic swab, which incorporates a breakpoint halfway down the shaft of the swab, and a Sigma Swab® tip (an open-celled foam bud) to capture the specimen (Figure 2). This specially designed tip is reported to provide optimum uptake and release of micro-organisms, and allow flow of the sample through the reagents used in the testing process, in comparison to a standard swab that uses either a cotton or rayon spun fibre tip.

The swab is then inserted into a plastic tube containing a viral transport medium (Figure 2). The manufacturer reports that this medium aids the survival of many types of common viruses, either at room temperature or refrigerated, stabilising the viral particles and utilising antimicrobial agents to prevent the growth of either bacteria or fungi that may also be present on the swab.^10^

The ENT-specific swab types are produced by Sigma for application in the nasopharynx.^10^ They utilise a finer tip and narrower shaft at the tip end of the swab. These swabs also incorporate a breakpoint feature.^10^

The breakpoint mechanism prevents contamination whilst transferring the shaft into the bottle; however, this feature makes the swab susceptible to fracture whilst still in the nasal cavity, as illustrated in our case. This situation could arise in non-cooperative patients, such as those with advanced dementia or severe learning difficulties, and in patients with anatomical variations (e.g. septal deviations and spurs). Head or limb movements can easily lead to the swab breaking and the swab tip being lost in the nasal cavity. If this does occur, further aerosol-generating procedures, such as flexible nasendoscopy and manual retrieval with surgical instruments, may be required in an already challenging patient.

### Foreign body removal

Foreign body removal from the nose is an aerosol-generating procedure and therefore warrants robust personal protective equipment, in line with ENT UK and NHS England guidance.^11,12^ Covid-19 precautions also include the examination and therapeutic intervention being delivered in a ‘one-stop’ episode, limiting the location, frequency and personnel to a single episode, to reduce the risk to the patient, nearby patients and healthcare staff.^11^

In order to optimise the success and retrieval of a nasal foreign body in a Covid-19 patient, and reduce the risk of aerosol generation, we recommend the application of a pledget soaked in vasoconstrictor and local anaesthetic solution to the nasal cavity, to reduce mucosal oedema and desensitise nasal mucosa prior to anterior rhinoscopy. Intranasal spray should be avoided, as this may induce aerosolisation.^13^

**Fig. 3.**
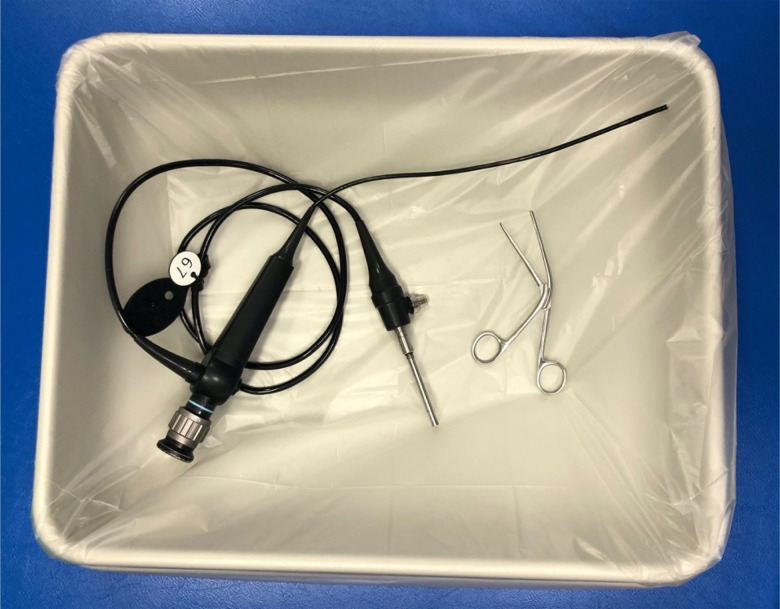
Flexible nasendoscope and Tilley's dressing forceps in tray.

Initially, anterior rhinoscopy may be carried out with a Thudicum nasal speculum, to accentuate the internal nasal valve area, the narrowest cross-sectional area of the nose. Foreign bodies deep to the internal valve require examination with a rigid or flexible nasendoscope. Close face-to-face proximity should be avoided where possible, and therefore nasal endoscopy should be carried out with a video display as per ENT UK guidance.^13^ Although the rigid scope is preferable, as it enables a single operator to handle both the scope and surgical instruments, it should be avoided in an uncooperative patient. A flexible nasendoscope requires two hands to operate and therefore an assistant is required for foreign body removal (Figure 3).

We opted to use a flexible nasendoscope with an assistant. Although this increased personnel exposure, we aimed to remove the swab at the patient's bedside in one single episode, to avoid the patient undergoing a general anaesthetic. Instrumentation to remove the foreign body is dictated by the item in question, and in this situation, we used Tilley's nasal dressing forceps to retrieve the nasal swab (Figure 3).

A retained foreign body post-procedure is a ‘never event’,^14^ and must be prevented at all costs. In a patient with a potential Covid-19 infection, a retained nasal swab not only poses a risk to the patient but also to healthcare staff, and the ensuing aerosol-generating procedures required to remove it further increase the risk of disease transmission to healthcare staff.

### Alternative test materials

The simplest way to avoid this complication is to utilise a product without a breakpoint. Based on the information from WHO^15^ and other sources,^16,17^ there are several alternative products for viral sampling that are readily available on the market, or already in use in the clinical environment, that do not have the breakpoint feature.

The use of alternative swabs, either dry or transported with a small amount of sterile saline, may provide a useful alternative in patients at risk of experiencing premature breaking of the viral swab shaft in the nasal cavity. This is supported by the WHO guidance, which suggests that nasopharyngeal and oropharyngeal swab products with Dacron® or polyester flocked swabs can be used, and if viral transport medium is unavailable, then sterile saline may be used in replacement.^15^ It must be noted that when there is likely to be a delay in specimens reaching the laboratory, the use of viral transport medium is strongly recommended by WHO.^15^

Druce *et al*. demonstrated that dry swabs, transported in either saline or dry, had similar efficacy in detecting various viruses as purpose-specific viral swabs that were sent in either universal transport medium or specific viral transport medium.^16^ Moore *et al*. revealed that the use of dry swabs with no transport medium was not only a suitable alternative, but it also produced a higher detection rate of respiratory viruses by using real-time nucleic acid sequence-based amplification, compared to more traditional methods of detection.^17^

• Viral swab sticks generally have a breakpoint feature
• Premature activation of the breakpoint in a complex group of patients can lead to a retained swab tip in the nasal cavity
• Nasal foreign body removal is an aerosol-generating procedure and requires robust personal protective equipment
• The simplest way to avoid this complication is to utilise a product without a breakpoint
• Alternative swab products with Dacron or polyester flocked tips can be used, and if viral transport medium is unavailable, then sterile saline may be used in replacement

We suggest that hospitals liaise with their local microbiology laboratory and seek alternative arrangements for swab testing if concerned about premature breakage in vulnerable patients.

## Conclusion

We have described a novel adverse event encountered with a Covid-19 viral swab. The patient was subjected to an aerosol-generating procedure to remove the nasal swab from the nasal cavity. The viral swab has an intrinsic design feature that gives rise to this complication. We have outlined appropriate techniques to address this complication and suggested a solution to circumvent this problem, which may be useful in a complex subset of suspected Covid-19 patients.

## References

[1] Kim JY, Ko JH, Kim Y, Kim YJ, Kim JM, Chung YS Viral load kinetics of SARS-CoV-2 infection in first two patients in Korea. J Korean Med Sci 2020;35:e86 32080991 10.3346/jkms.2020.35.e86 PMC7036338

[2] Zhou F, Yu T, Du R, Fan G, Liu Y, Liu Z Clinical course and risk factors for mortality of adult inpatients with COVID-19 in Wuhan, China: a retrospective cohort study. Lancet 2020;395:1054–62 32171076 10.1016/S0140-6736(20)30566-3 PMC7270627

[3] World Health Organization. Virtual press conference on COVID-19–11 March 2020. In: https://www.who.int/docs/default-source/coronaviruse/transcripts/who-audio-emergencies-coronavirus-press-conference-full-and-final-11mar2020.pdf?sfvrsn=cb432bb3_2 [6 July 2020]

[4] Kelly-Cirino CD, Nkengasong J, Kettler H, Tongio I, Gay-Andrieu F, Escadafal C Importance of diagnostics in epidemic and pandemic preparedness. BMJ Glob Health 2019;4:e001179 10.1136/bmjgh-2018-001179 PMC6362765 30815287

[5] Zou L, Ruan F, Huang M, Liang L, Huang H, Hong Z SARS-CoV-2 viral load in upper respiratory specimens of infected patients. N Engl J Med 2020;382:1177–9 32074444 10.1056/NEJMc2001737 PMC7121626

[6] Liu Y, Yan LM, Wan L, Xiang TX, Le A, Liu JM Viral dynamics in mild and severe cases of COVID-19. Lancet Infect Dis 2020;20:656–7 32199493 10.1016/S1473-3099(20)30232-2 PMC7158902

[7] Pang J, Wang MX, Ang IYH, Tan SHX, Lewis RF, Chen JI Potential rapid diagnostics, vaccine and therapeutics for 2019 novel coronavirus (2019-nCoV): a systematic review. J Clin Med 2020;9:E623 32110875 10.3390/jcm9030623 PMC7141113

[8] Rodriguez-Morales AJ, Cardona-Ospina JA, Gutiérrez-Ocampo E, Villamizar-Peña R, Holguin-Rivera Y, Escalera-Antezana JP Clinical, laboratory and imaging features of COVID-19: a systematic review and meta-analysis. Travel Med Infect Dis 2020;34:101623 32179124 10.1016/j.tmaid.2020.101623 PMC7102608

[9] Yu F, Yan L, Wang N, Yang S, Wang L, Tang Y Quantitative detection and viral load analysis of SARS-CoV-2 in infected patients. Clin Infect Dis 2020. Epub 2020 Mar 28 10.1093/cid/ciaa345 PMC7184442 32221523

[10] MWE Medical Wire. Sigma Virocult – Virus Specimen Transport for Molecular and Culture Techniques. In: https://www.mwe.co.uk/modules/downloadable_files/assets/sigma-virocult.pdf [6 July 2020]

[11] ENT UK. Guidance PPE for patients with emergency oropharyngeal and nasopharyngeal conditions whose COVID status is unknown. In: https://www.entuk.org/sites/default/files/BAOMS%20ENT%20COVID%20Advice%20Update%2025%20March%202019%20Final.pdf [6 July 2020]

[12] Public Health England. COVID-19: personal protective equipment use for aerosol generating procedures. In: https://www.gov.uk/government/publications/covid-19-personal-protective-equipment-use-for-aerosol-generating-procedures [6 July 2020]

[13] ENT UK. Nasal endoscopy and laryngoscopy examination of ENT patients. In: https://www.entuk.org/nasal-endoscopy-and-laryngoscopy-examination-ent-patients [23 March 2020]

[14] NHS England and NHS Improvement. Provisional Publication of Never Events Reported as Occurring Between 1 April 2019 and 29 February 2020. London: NHS England and NHS Improvement, 2020;10

[15] World Health Organization. Laboratory Testing for Coronavirus Disease 2019 (COVID-19) in Suspected Human Cases: Interim Guidance, 2 March 2020. Geneva: World Health Organization, 2020;6

[16] Druce J, Garcia K, Tran T, Papadakis G, Birch C. Evaluation of swabs, transport media, and specimen transport conditions for optimal detection of viruses by PCR. J Clin Microbiol 2012;50:1064–5 22205810 10.1128/JCM.06551-11 PMC3295134

[17] Moore C, Corden S, Sinha J, Jones R. Dry cotton or flocked respiratory swabs as a simple collection technique for the molecular detection of respiratory viruses using real-time NASBA. J Virol Methods 2008;153:84–9 18761378 10.1016/j.jviromet.2008.08.001

